# How do I get the most out of my protein sequence using bioinformatics tools?

**DOI:** 10.1107/S2059798321007907

**Published:** 2021-08-24

**Authors:** Joana Pereira, Vikram Alva

**Affiliations:** a Department of Protein Evolution, Max Planck Institute for Developmental Biology, Max-Planck-Ring 5, 72076 Tübingen, Germany

**Keywords:** deep homology searches, protein sequence annotation, homology modeling, coiled coils, sequence features, bioinformatics tools

## Abstract

Biochemical and biophysical experiments are essential for uncovering the three-dimensional structure and biological role of a protein of interest. However, meaningful predictions can frequently also be made using bioinformatics resources that transfer knowledge from a well studied protein to an uncharacterized protein based on their evolutionary relatedness. These predictions are helpful in developing specific hypotheses to guide wet-laboratory experiments. Commonly used bioinformatics resources include methods to identify and predict conserved sequence motifs, protein domains, transmembrane segments, signal sequences, and secondary as well as tertiary structure. Here, several such methods available through the MPI Bioinformatics Toolkit (https://toolkit.tuebingen.mpg.de) are described and how their combined use can provide meaningful information on a protein of unknown function is demonstrated. In particular, the identification of homologs of known structure using *HHpred*, internal repeats using *HHrepID*, coiled coils using *PCOILS* and *DeepCoil*, and transmembrane segments using *Quick*2*D* are focused on.

## Abstract 2

Bioinformatics tools, primarily those available through the MPI Bioinformatics Toolkit, for the annotation of protein sequences are described. These include tools for the identification of homologs of known structure, protein domains, sequence repeats, coiled coils, transmembrane segments and signal sequences.

## 1. Introduction   

With a protein sequence of interest at hand, life scientists aim at obtaining all possible information towards uncovering its biological role. Wet-laboratory experiments are fundamental to such a task. However, the increasing availability of protein sequence, structural and functional data has allowed the development of multiple computational resources that help to make informative predictions to guide experiments. These resources include methods for detecting homologs in protein sequence and structure databases, detecting sequence features such as repeats, coiled coils, transmembrane segments, signal sequences and secondary structures, and predicting three-dimensional structures. Their combined results generally help to answer various questions regarding a protein of interest, including (i) which domains may be present, (ii) what its cellular localization may be, (iii) which segments may be fibrous and be responsible for its function or impair some experimental steps and (iv) which molecular functions may be expected.

Bioinformatics tools for protein sequence analysis have been developed over more than 30 years by groups worldwide, and their list is so extensive that choosing the most suitable and performant ones to use can often be overwhelming. Integrative web resources, where multiple best-performing tools are available within the same platform, provide a great solution. Examples include the EMBL–EBI Bioinformatics Web Services (Madeira *et al.*, 2019 ▸), the SIB Bioinformatics Resource Portal (SIB Swiss Institute of Bioinformatics Members, 2016 ▸), the National Center for Biotechnology Information Web Resources (NCBI Resource Coordinators, 2018 ▸), the PredictProtein server (Bernhofer *et al.*, 2021 ▸) and the Max Planck Institute (MPI) Bioinformatics Toolkit (Zimmermann *et al.*, 2018 ▸).

The MPI Bioinformatics Toolkit (https://toolkit.tuebingen.mpg.de/) was launched in 2005 to provide life scientists with easy, web-based access to the best-performing bioinformatics tools and databases. It currently includes 36 in-house and external tools for (i) sequence-similarity searching, (ii) sequence-repeat detection and (iii) sequence-feature prediction, including that of secondary structure, disordered regions, coiled-coil regions, transmembrane segments and signal sequences. The Toolkit also offers easy, web-based access to *HHblits* and *HHpred* (Steinegger *et al.*, 2019 ▸), two of the most sensitive tools for the detection of remote evolutionary relationships. Because of the popularity of these two tools, the Toolkit has established itself as an important integrative resource for molecular-biology research.

Here, we provide practical tips for using some of the main tools available within the Toolkit; for comprehensive protocols, please refer to Gabler *et al.* (2020 ▸). To demonstrate the different steps involved in the annotation of an uncharacterized protein, we use a metagenome-derived hypothetical protein, EHM23_20970 (EntrezID RPJ57313.1), thought to originate from an acidobacterium, as an example. This protein was recently predicted to contain a β-propeller domain of the VCBS superfamily, but its biological role is currently unknown (Pereira & Lupas, 2021 ▸).

## 2. Homology searches   

When analyzing an uncharacterized protein sequence of interest, the first step is to identify functionally or structurally characterized homologs in protein sequence and structure databases such as the nonredundant database (nr) at NCBI or the Protein Data Bank (PDB). This helps in the inference of function and the modeling of three-dimensional structures through extrapolation by homology. Common sequence-search methods include (i) *BLASTp* (Altschul *et al.*, 1997 ▸; Ladunga, 2017 ▸), which compares a single sequence with a sequence database, (ii) *PSI-BLAST* (Altschul *et al.*, 1997 ▸), which compares a position-specific scoring matrix (PSSM), also commonly referred to as a profile, with a sequence database, (iii) *HMMER* (Prakash *et al.*, 2017 ▸; Potter *et al.*, 2018 ▸), which compares a profile hidden Markov model (HMM) with a sequence database, and (iv) *HHsearch* (Steinegger *et al.*, 2019 ▸; Söding, 2005 ▸), which compares a profile HMM with a profile HMM database. Due to their different underlying approaches, each of these methods has different sensitivities and detects homologous relationships at different evolutionary distances. These four tools are available through the ‘Search’ section of the Toolkit and allow searches in various sequence databases.

By comparing single sequences, *BLASTp* searches for close homologs of a query protein sequence. The horizon of its search can be expanded further by iteration using *PSI-BLAST* (Altschul *et al.*, 1997 ▸), wherein the multiple sequence alignment (MSA) of the matches in one round is used to build a PSSM that captures the conservation pattern in the alignment and records it as a matrix of scores for each position in the alignment. This PSSM is used in the next round to detect new matches, and after each round it is updated with the newly detected matches, allowing a continuous expansion of the sampled sequence space until no new homologs are found.

*HMMER*, on the other hand, compares a profile HMM, another statistical description of the conservation pattern of a sequence alignment, with a database of protein sequences. As in PSSMs, for each column in an MSA, the equivalent column in the corresponding HMM contains the probability of occurrence for each of the 20 amino acids; the difference lies in the presence of four additional probabilities that describe how often amino acids are inserted and deleted at that position. With this, *HMMER* evaluates the probability of a database sequence containing the sequence pattern of a given profile HMM and can often detect more distant evolutionary relationships. Like *BLAST*, its search horizon can be expanded by iteration, as implemented in *JackHMMER* (not available through the Toolkit; https://www.ebi.ac.uk/Tools/hmmer/search/jackhmmer; Johnson *et al.*, 2010 ▸).

*HHsearch* and its accelerated and iterative version *HHblits* (Remmert *et al.*, 2012 ▸) achieve a further increase in sensitivity by comparing profile HMMs with a database of profile HMMs and by incorporating secondary-structure information in the underlying profile HMMs, either as predicted by *PSIPRED* (Jones, 1999 ▸) or assigned from a 3D structure by *DSSP* (Touw *et al.*, 2015 ▸; Kabsch & Sander, 1983 ▸). They are currently the most sensitive methods for detecting distant evolutionary relationships that typically remain undetected by other search methods. *HHsearch* and *HHblits* are therefore at the core of multiple state-of-the-art structure-prediction workflows, from template-based methods such as *HHpred* (Hildebrand *et al.*, 2009 ▸; Zimmermann *et al.*, 2018 ▸) and *SWISS-MODEL* (Waterhouse *et al.*, 2018 ▸) to *ab initio* contact-based methods such as *AlphaFold* (Senior *et al.*, 2020 ▸; Jumper *et al.*, 2021 ▸), *trRosetta* (Yang, Anishchenko *et al.*, 2020 ▸) and *RoseTTAFold* (Baek *et al.*, 2021 ▸).

## 3. *HHpred* for sensitive protein-homology detection and structure prediction   

The most widely used tool within the Toolkit is *HHpred*, a server for protein-domain annotation and structure prediction based on *HHsearch* (Hildebrand *et al.*, 2009 ▸; Gabler *et al.*, 2020 ▸; Zimmermann *et al.*, 2018 ▸). Starting from an input protein sequence, *HHpred* builds an MSA and generates a profile HMM. By default, MSA generation is carried out with three iterations of *HHblits* over the UniRef clusters database filtered for a maximum pairwise sequence identity of 30% (UniRef30; Mirdita *et al.*, 2017 ▸). The number of iterations, the *E*-value cutoff for sequence inclusion and the search method itself can be changed depending on how deep the user desires the MSA to be. If *PSI-BLAST* is used for this step, sequence searches are carried out against the nr protein-sequence database filtered for a maximum sequence identity of 70% (nr70; Zimmermann *et al.*, 2018 ▸).

The calculated profile HMM is then searched against user-selected profile HMM databases. By default, the Protein Data Bank filtered for a maximum pairwise sequence identity of 70% (PDB70) is searched, but several other databases are also offered, including the Structural Classification of Proteins (Chandonia *et al.*, 2019 ▸) and the Evolutionary Classification of Protein Domains (Cheng *et al.*, 2014 ▸) databases filtered for a maximum sequence identity of 70% (SCOPe70 and ECOD_F70, respectively), Pfam-A (Mistry *et al.*, 2020 ▸) and the NCBI database of Conserved Domains (CD; Yang, Derbyshire *et al.*, 2020 ▸). Presently, up to four databases can be searched at a time. While searches against the PDB70 database allow the identification of homologs of known structure that may be used for homology modeling, searches against domain databases aid in the identification and annotation of putative domain regions and the inference of function. Additionally, *HHpred* also offers profile HMM databases for several representative archaeal, bacterial and eukaryotic proteomes (Zimmermann *et al.*, 2018 ▸).

**Figure 1:**
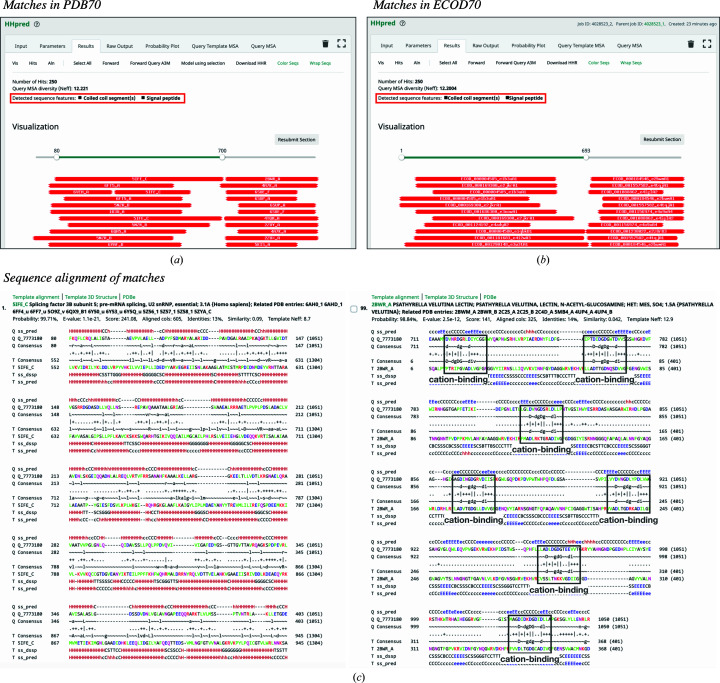
Identification of homologs of known structure for the hypothetical protein EHM23_20970 using *HHpred*. Output pages for searches against the (*a*) PDB70 and (*b*) ECOD70 (ECOD_F70) profile HMM databases are shown. The alert message displayed when coiled-coiled segments and/or signal peptides are predicted is highlighted by a red box. (*c*) Sequence alignments for the best match for the N- and C-terminal regions. The sevenfold repetition of a conserved putative cation-binding motif in the C-terminal region is highlighted.

Upon completion of the search, the results page provides three outputs: (i) a visual summary of the matches color-coded by their *HHsearch* probability [red (100%) to blue (20%)] (Figs. 1 ▸ *a* and 1 ▸ *b*), (ii) a table summarizing the matches and (iii) pairwise query–template alignments (Fig. 1 ▸ *c*). The matches are sorted by their *HHpred* probability value and by default only the top 250 matches are displayed, but a maximum of 10 000 can be shown. Most representations on the results page are interactive; for example, clicking on a match in the visual summary takes the user to the corresponding alignment. Before selecting any match for downstream analysis (for example homology modeling or further sequence searches), it is advisable to analyze the corresponding alignment for conserved sequence motifs (Fig. 1 ▸ *c*) or important deletions or insertions to make the most informed predictions or manual selection of templates. For detailed information on various search parameters and on understanding the search results, please refer to Gabler *et al.* (2020 ▸).

**Figure 2:**
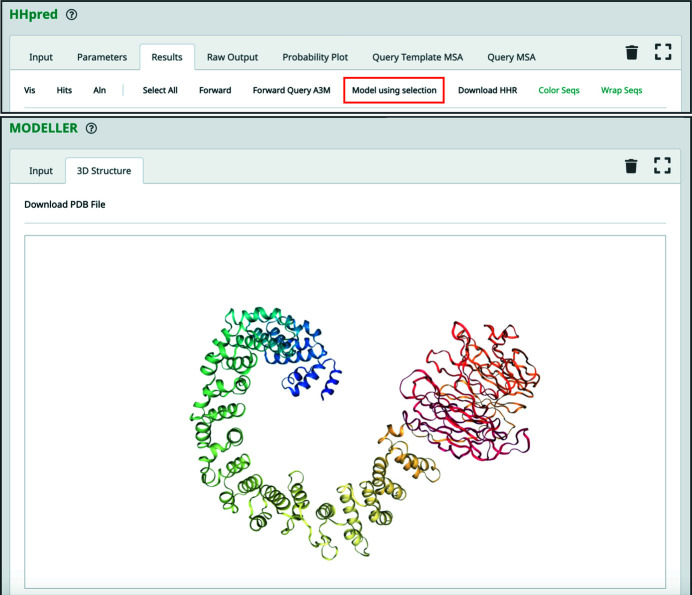
Homology modeling of the hypothetical protein EHM23_20970. In the top panel, a screenshot of the *HHpred* results page header is shown, highlighting the option ‘Model using selection’. The top match for each domain in EHM23_20970 was selected (PDB entries 5ife chain *C* and 2bwr chain *A*) and forwarded to *MODELLER*. The resulting model is shown in the bottom panel.

After careful inspection of the query–template alignments, manually selected alignments from a search against the PDB70 database can be forwarded to *MODELLER* (Webb & Sali, 2021 ▸) for homology modeling; that is, for building a structural model of the query protein sequence by using the match as a template (Fig. 2 ▸). This can be achieved by clicking ‘Model using selection’, which will start a new Toolkit job, generating an alignment in PIR format to be forwarded as input to *MODELLER*; if necessary, this alignment can be manually adjusted before starting the *MODELLER* job. Users with a precomputed *HHpred* query–template alignment in PIR format can also run *MODELLER* directly from the ‘3ary Structure’ section. As with any method for homology modeling, some important considerations should be taken into account: (i) errors in the alignment will introduce errors in the model, and the lower the sequence similarity between the query and the template, the higher the probability of such errors will be, (ii) side-chain placement becomes unreliable at sequence identities below 70% and (iii) as no dedicated loop-modeling tool is employed, long loops for which no templates are available are not modeled reliably. For these reasons, before any downstream application of the calculated model its quality should be evaluated and, if necessary, it should be refined; for a detailed review of this topic, please refer to Haddad *et al.* (2020 ▸).

Often, *HHpred* searches may not identify homologs for specific regions of the input sequence. The reasons for this could be manifold: (i) the region may not have any homologs of known structure, (ii) it may correspond to an intrinsically disordered region or (iii) it may be a highly diverged form of a known domain. In such cases, it is typically helpful to re-run the search for that region alone. Highly conserved sequences tend to bias the profile HMM by contributing a high number of homologs, down-weighting less conserved regions and making them undetectable. By running *HHpred* with the region of interest alone, the profile HMM will not be biased by its flanking regions in the full-length sequence.

The following recommendations are made.

(i) When only very close homologs are to be found, set the ‘MSA generation iterations’ to 0.
(ii) Set ‘Max target hits’ to 10 000 to obtain a more comprehensive set of matches.
(iii) To compare two sequences or MSAs, use the pairwise mode of *HHpred*; click on the switch labeled ‘Align two sequences/MSAs’ located below the input textbox to activate it.
(iv) When an *HHpred* search yields no matches for certain regions of a protein, re-run the searches with those regions alone.
(v) Always inspect the alignments for conserved sequence motifs. In particular, check the row between the query and template consensus sequences for clusters of three or more matching columns (marked by a ‘|’ sign). Check whether the identified conserved motifs have a characterized function in homologs detected by *HHpred*.
(vi) Always check the quality of a homology-based model before any downstream application.

## 4. Repeat detection   

In addition to identifying experimentally characterized homologs in sequence and structure databases, it is often also helpful to detect internal sequence repeats in the protein sequence of interest. More than 14% of all proteins, and as many as 25% in eukaryotes, are predicted to contain internal sequence repeats (Marcotte *et al.*, 1999 ▸), which often correspond to structural or functional units (Andrade, Perez-Iratxeta *et al.*, 2001 ▸; Söding & Lupas, 2003 ▸). Therefore, their identification provides clues about the domain organization, fold and function of proteins, especially of those without any homologs of known structure and function. Additionally, it may help to gain insights into possible fibrous, elongated or symmetric segments of the protein that may affect its experimental characterization. In the specific case of macromolecular crystallography, local structural symmetry is a source of noncrystallographic symmetry (NCS), which can be both a valuable asset (Kleywegt, 1996 ▸; Terwilliger, 2002 ▸) and a complication (Ruf *et al.*, 2016 ▸; Jamshidiha *et al.*, 2019 ▸) in crystallographic structure determination.

In the MPI Toolkit, seven different methods for detecting internal repeats in protein sequences are available through the ‘Sequence Analysis’ section. *REPPER* (Gruber *et al.*, 2005 ▸) performs Fourier transform and internal homology analysis to detect short, gapless repeats of all possible periodicities within a range of two to 100 residues. *TPRpred* (Karpenahalli *et al.*, 2007 ▸) uses pre-computed profile HMMs to detect tetratricopeptide repeats (TPRs), pentatricopeptide repeats (PPRs) and Sel1-like repeats (SLRs). *PCOILS* (Gruber *et al.*, 2006 ▸), *MARCOIL* (Delorenzi & Speed, 2002 ▸), *DeepCoil* (Ludwiczak *et al.*, 2019 ▸) and *DeepCoil*2 use different approaches to detect coiled-coil segments, whereas *HHrepID* (Biegert & Söding, 2008 ▸) is an automated method for the *de novo* identification of repeats.

## 5. *HHrepID* for *de novo* repeat detection   

**Figure 3:**
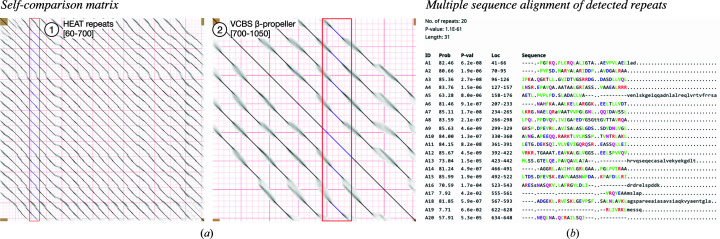
Detection of repeats in the hypothetical protein EHM23_20970 using *HHrepID*. For the analysis, the sequence of each domain detected in EHM23_20970 with *HHpred* was used as input for two separate *HHrepID* jobs. (*a*) The self-comparison matrices for the two domains are shown. The boundaries of the domains are indicated within square brackets. Sequence repeats detected at a *p*-value threshold of 1 × 10^−1^ are shown as blue lines within a red box. (*b*) The multiple sequence alignment of the HEAT repeats detected in domain 1 at a *p*-value cutoff of 1 × 10^−1^ [represented by blue lines in (*a*)] are shown. The probability, *p*-value and the boundaries of the detected repeats are indicated.

*HHrepID* employs HMM–HMM comparison for the *de novo* detection of highly divergent tandem repeats in protein sequences (Biegert & Söding, 2008 ▸; Remmert *et al.*, 2010 ▸). It starts by generating a profile HMM for the input sequence using three iterations of *HHblits* over the UniRef30 database. Next, it uses HMM–HMM self-comparison to search for local suboptimal alignments and to detect sequence signatures of repeats. The result is a graphical representation of the self-comparison matrix, where entries (*i*, *j*) correspond to the probability of residue *i* being aligned with residue *j*, and a multiple sequence alignment of the repeat units found by analyzing the matrix (including their significance value and boundaries; Fig. 3 ▸).

A repeat sequence is considered to be significant if the self-alignment *p*-value is below a given threshold (1 × 10^−1^ by default) and is highlighted in the self-comparison matrix as a blue line. However, highly divergent repeats may not pass this threshold and may not be included in the alignment, but signals for them may still be observed in the matrix as dark or light gray lines (Fig. 3 ▸ *a*). Such divergent repeats can be found at the termini, between detected repeat-containing regions or even as long linkers. Therefore, it is always helpful to analyze the self-comparison matrix and the linkers between repeats for divergent repeats. If the linkers are about the same size as the detected repeats, they may represent degenerate forms. In such cases, it is advisable to realign the automatically detected and manually included repeats with multiple sequence alignment tools (available through the ‘Alignment’ section).

*HHrepID* works best with protein sequences containing a single domain or a single repeat type. Ideally, sequences should be between 100 and 300 residues long. While shorter sequences usually gather too few homologs in the MSA generation step, longer sequences may contain multiple domains or different types of repeats. *HHpred* could be used first to detect domains, and subsequently *HHrepID* could be run on the individual domains. Also, if an *HHrepID* job yields repeats of different types, the detected repeats could be refined by re-running *HHrepID* with sequence segments corresponding to one repeat type.

The following recommendations are made.

(i) Carry out an *HHpred* search against a database of domains (for example SCOPe, ECOD or Pfam-A) and subsequently run *HHrepID* for each domain individually.
(ii) Pay attention to linker regions between repeats. If they are about the same length as the detected repeats, they may represent highly divergent repeats that scored below the significance threshold.
(iii) When analyzing an alignment of repeats yielded by *HHrepID*, it can be useful to realign them using other sequence-alignment tools (for example *MSAProbs*; Liu *et al.*, 2010 ▸).

## 6. Coiled-coil prediction   

Coiled coils are a ubiquitous class of repetitive protein segments that support various biological roles from transport to structural rigidity and signal transduction (Lupas & Bassler, 2017 ▸). They consist of two or more α-helices that wind around each other in a parallel or antiparallel orientation to form a superhelical bundle. The bundle is held together by a primarily hydrophobic core following a ‘knobs-into-holes’ packing (Lupas *et al.*, 2017 ▸). Canonical coiled coils are characterized by a seven-residue sequence repeat (the ‘heptad’), where each position is labeled *a*–*g*; the residues at positions *a* and *d* are usually oriented towards the core and are primarily hydrophobic. However, coiled coils with other periodicities are also known and are referred to as ‘noncanonical’ coiled coils. These are described as combinations of three- and four-residue sequence segments (for example an 11-residue repeat, or hendecad, is the result of the combination of 3 + 4 + 4 segments), and their packing deviates from the knobs-into-holes geometry. The MPI Toolkit offers four tools for the prediction of coiled-coil regions from sequence alone: *PCOILS* (Gruber *et al.*, 2006 ▸; Lupas *et al.*, 1991 ▸), *MARCOIL* (Delorenzi & Speed, 2002 ▸), *DeepCoil* (Ludwiczak *et al.*, 2019 ▸) and *DeepCoil*2.

*PCOILS* detects coiled-coil segments in a protein sequence or an MSA using sequence–profile or profile–profile comparisons. The Toolkit implementation of *PCOILS* allows the user to run the predictions on a protein sequence, a custom MSA or an MSA built internally by the Toolkit. Additionally, the user can set two parameters: the profile matrix (MTIDK, MTK, PDB or Iterated) and a weighting option for core residues (yes or no). The MTIDK and MTK matrices are based on myosins, paramyosins, tropomyosins, intermediate filaments type IV, desmosomal proteins and kinesins (Lupas *et al.*, 1991 ▸), whereas the PDB and Iterated matrices are based on larger coiled-coil data sets derived from the PDB and the nr database, respectively. The Iterated matrix performs the best and the MTK matrix the worst when the prediction is carried out only using the input sequence alone. However, when the predictions are carried out using an MSA, all matrices perform similarly.

**Figure 4:**
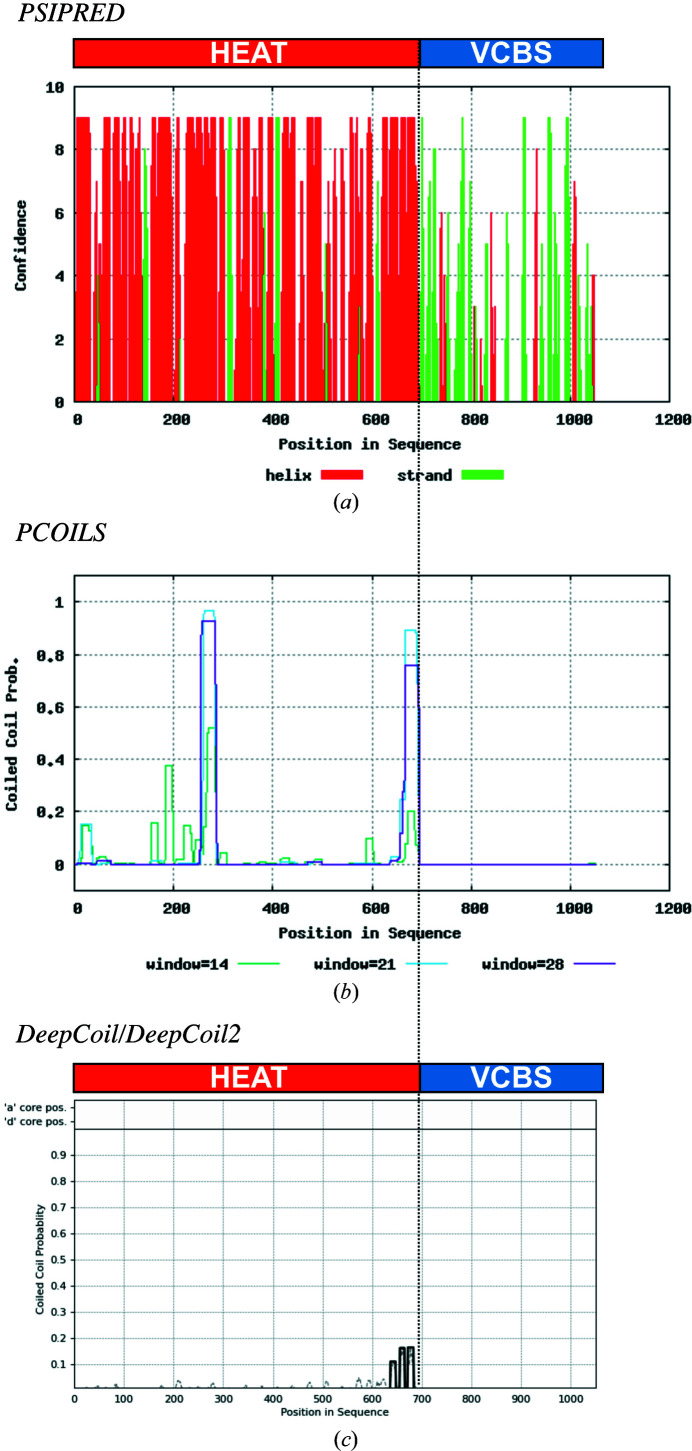
Coiled-coil prediction for the hypothetical protein EHM23_20970 using *PCOILS* and *DeepCoil*2. The graphical output of each tool, including the secondary-structure prediction carried out by *PCOILS* with *PSIPRED*, is shown and aligned to provide a comparison.

As coiled coils are typically fibrous and solvent-exposed, all but the core positions (*a* and *d*) have a high probability of being occupied with hydrophilic residues. Consequently, since all positions are weighted equally in the unweighted mode of *PCOILS*, highly charged hydrophilic sequences are often predicted to be coiled coils, even in the absence of heptad periodicity. This issue can be resolved using the weighted mode of *PCOILS*, which assigns the same weight to the two hydrophobic positions *a* and *d* as to the five hydrophilic positions *b*, *c*, *e*, *f* and *g* (2.5:1 in the weighted mode versus 1:1 in the unweighted mode). *PCOILS* proceeds by comparing the input sequence or MSA with the user-selected matrix using sliding windows of three different sizes (14, 21 and 28 residues, corresponding to two, three and four heptads, respectively). The output is the coiled-coil-forming probability and frame (*a*–*g*) for each residue in the input sequence; as a rule of thumb, residues with probabilities above 50% can be considered to be part of a coiled-coil segment. In the MPI Toolkit, these probabilities are made available through a table and a graph, shown together with the secondary-structure prediction carried out with *PSIPRED* (Jones, 1999 ▸; Figs. 4 ▸ *a* and 4 ▸ *b*).

Unlike *PCOILS*, *MARCOIL* is a windowless HMM-based tool for the detection of canonical coiled-coil regions. Its output is similar to that of *PCOILS*, with a graphical representation of the posterior probabilities along the sequence and a probability list with the corresponding predicted per-residue heptad frame. The performance of *MARCOIL* is comparable to that of *PCOILS*, but it is extremely sensitive to highly charged false positives.

*DeepCoil* and its updated version *DeepCoil*2 are the most recent addition to the Toolkit’s repertoire of coiled-coil prediction methods. *DeepCoil* is a neural network-based method trained on more than 10 400 nonredundant canonical coiled coils of known structure. It detects both canonical and noncanonical coiled coils, including many that are undetectable with *PCOILS* and *MARCOIL*. *DeepCoil* predictions can be carried out based on a single sequence or an MSA provided by the user or built by the Toolkit using three iterations of *PSI-BLAST* over the nr90 database. While *DeepCoil* uses PSSMs generated by *PSI-BLAST* to capture evolutionary information, *DeepCoil*2 uses the pre-trained protein language model SeqVec that is based on the ELMo language model from the domain of natural language processing (Heinzinger *et al.*, 2019 ▸; Peters *et al.*, 2018 ▸). The output of *DeepCoil* is similar to that of *PCOILS* and *MARCOIL*, with a graphic summary of the predictions (Fig. 4 ▸ *b*) and a text output with per-residue probability values; *DeepCoil*2 also predicts the heptad registers.

The decision on which of these methods to use is a trade-off between required runtime and accuracy. *PCOILS* and *MARCOIL* are extremely fast and good at predicting canonical coiled coils (Ludwiczak *et al.*, 2019 ▸; Gruber *et al.*, 2006 ▸; Li *et al.*, 2016 ▸). However, they often assign high coiled-coil probabilities to highly charged sequences. *DeepCoil* and *DeepCoil*2 are slower but are more accurate and can detect noncanonical coiled coils.

The following recommendations are made.

(i) When using *PCOILS*, the probabilities assigned using a 28-residue window are best suited for detecting new coiled-coil regions in a protein of interest, whereas 14-residue windows are good for defining boundaries and heptad registers of detected coiled coils.
(ii) Since *PCOILS* is biased towards highly charged sequences, predictions should be made and compared using the weighted and unweighted modes and corroborated further using *DeepCoil*2.

## 7. Integrative annotation of sequence features   

Sequence features are segments that confer specific characteristics on a protein and are important for its function or structure. These include not only domains, short sequence motifs and repeats, but also secondary structure, intrinsically disordered regions, transmembrane segments and signal sequences. While the prediction of secondary structure and intrinsically disordered regions provides information complementary to the annotations carried out with *HHpred* and repeat detection, their annotation is especially important when no homology is found to any protein of known structure. Transmembrane segments and signal sequences, on the other hand, provide additional information regarding the possible cellular localization of a protein of interest.

**Figure 5:**
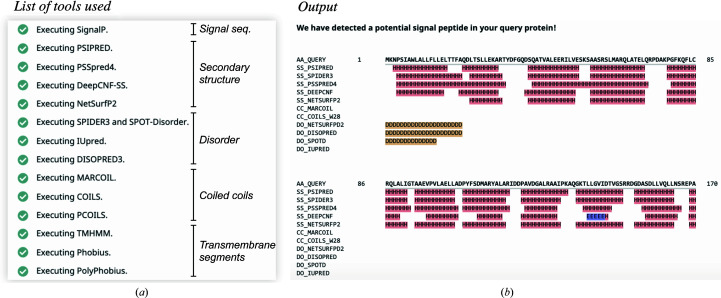
Sequence-feature annotation in the hypothetical protein EHM23_20970 using *Quick*2*D*. (*a*) The list of tools executed by *Quick*2*D*, depicting their target features. (*b*) Example output for the first 170 residues, highlighting its all-helical propensity and the presence of a putative signal peptide and an intrinsically disordered N-terminal segment.

The Toolkit includes a meta-tool, *Quick*2*D* (Zimmermann *et al.*, 2018 ▸; Gabler *et al.*, 2020 ▸), for analyzing sequence features. *Quick*2*D* runs several feature-prediction tools and presents their results in a single view (Fig. 5 ▸). These tools include (Fig. 5 ▸ *a*) *PSIPRED* (Jones, 1999 ▸) and *NetSurfP*-2.0 (Klausen *et al.*, 2019 ▸) for the prediction of secondary structure, *SPIDER*3 (Heffernan *et al.*, 2018 ▸) and *DISOPRED3* (Jones & Cozzetto, 2015 ▸) for the prediction of intrinsically disordered regions, *TMHMM* (Krogh *et al.*, 2001 ▸) and *Phobius* (Käll *et al.*, 2004 ▸) for the prediction of α-helical transmembrane segments, *SignalP* (Almagro Armenteros *et al.*, 2019 ▸) for the identification of potential N-terminal signal peptides and *PCOILS* and *MARCOIL* for the identification of coiled-coil regions.

If a signal sequence is detected, a notification is displayed at the top of the output page. Additionally, if the first 20–35 residues of a protein are predicted to be disordered (Fig. 5 ▸ *b*), it is highly likely to be a signal peptide. However, while *SignalP* also predicts the potential secretory pathway that a query protein is targeted to based on whether it originates from a eukaryote, an archaeon or a Gram-positive or Gram-negative bacterium (Almagro Armenteros *et al.*, 2019 ▸), *Quick*2*D* does not display such information. Similarly, while *TMHMM* and *Phobius* also predict the topology of membrane segments, *Quick*2*D* does not. Such information could be obtained using the *SignalP*, *THHMM* or *Phobius* servers directly or using the *TOPCONS* metaserver (Tsirigos *et al.*, 2015 ▸; https://topcons.net), which runs several methods for the prediction of transmembrane α-helices. We note that all search tools with the Toolkit (for example *HHpred*) also display a message if coiled coils, signal peptides or transmembrane segments are detected (Figs. 1 ▸ *a* and 1 ▸ *b*).

Quick2D does not predict transmembrane β-strands, such as those found in outer membrane β-barrels (OMBBs). OMBBs can be predicted using the HMM-based tool *HHomp* (Remmert *et al.*, 2009 ▸) within the Toolkit, or using the external servers *BOCTOPUS*2 (Hayat *et al.*, 2016 ▸) or *BetAware-Deep* (Madeo *et al.*, 2020 ▸).

The following recommendations are made.

(i) In order to obtain further insights about a sequence predicted to contain a signal peptide or α-helical transmembrane segments, re-run the prediction again using a dedicated server such as *SignalP*, *TMHMM*, *Phobius* or *TOPCONS*.
(ii) If a notification concerning the prediction of a signal peptide is displayed and the N-terminal part, *i.e.* the first 20–35 residues, is predicted to be disordered, it is likely to correspond to the detected signal sequence.
(iii) Always inspect the output of search tools (for example *HHpred*) within the Toolkit for notifications regarding the presence of sequence features.

## 8. Example: annotation of the hypothetical protein EHM23_20970   

The hypothetical protein EHM23_20970 (EntrezID RPJ57313.1) is a 1051-residue putative protein derived from a sediment metagenome that was obtained from the sequencing of environmental samples collected at the North Dakota Cottonwood Lake Study Area and the Prairie Pothole Region wetland (Dalcin Martins *et al.*, 2018 ▸). EHM23_20970 is thought to originate from an acidobacterium, and we came across it while studying the evolutionary relationships between the β-propeller domains in α-integrin, tachylectin-2 and proteins of the VCBS superfamily (Pereira & Lupas, 2021 ▸). Its β-propeller domain formed a distinct group with the β-propellers of several other hypothetical proteins.

A *BLASTp* search over the nr database (version of January 2021) resulted in 5002 hits to hypothetical proteins at an *E*-value cutoff of 10^−3^. While ten of these hits made full-length matches, the rest only matched its C-terminal segment (residues 700–1051) which, as we will see in the following, corresponds to its β-propeller domain. Running three iterations of *PSI-BLAST* yielded the same result, suggesting that while EHM23_20970 is not a singleton, it is also not a close homolog of any hitherto characterized protein. Similarly, when we searched for homologs of known structure in PDB70 (version of January 2021) with *HHpred*, no full-length match was found (Fig. 1 ▸ *a*). However, the obtained matches indicated the presence of two distinct regions: an N-terminal region from residues 60 to 700 and a C-terminal region from residues 711 to 1050. The best match to the N-terminal region was made by an all-α-helical region of human splicing factor 3B subunit 5 (PDB entry 5ife, chain *C*; probability 99.71%) and that to the C-terminal region was made by an all-β-fold lectin (PVL) from *Psathyrella velutina* (PDB entry 2bwr, chain *A*; probability 98.84%) (Fig. 1 ▸ *c*). A subsequent *HHpred* search against the ECOD70 domain database indicated that the N-terminal region contains HEAT repeats (Jernigan & Bordenstein, 2015 ▸; Andrade, Petosa *et al.*, 2001 ▸) and the C-terminal region contains a VCBS-like β-propeller domain (Fig. 1 ▸ *c*). Both of these domains are repetitive: while the HEAT repeat comprises two α-helices connected by a short linker and is typically tandemly repeated 3–36 times to form open-ended solenoids, β-propellers are toroids built of 4–12 four-stranded β-meanders. By forwarding the aforementioned best-scoring templates, PDB entry 5ife chain *C* and PDB entry 2bwr chain *A*, to *MODELLER*, we built a preliminary full-length model of EHM23_20970 (Figs. 2 ▸ and 6 ▸ *b*).

**Figure 6:**
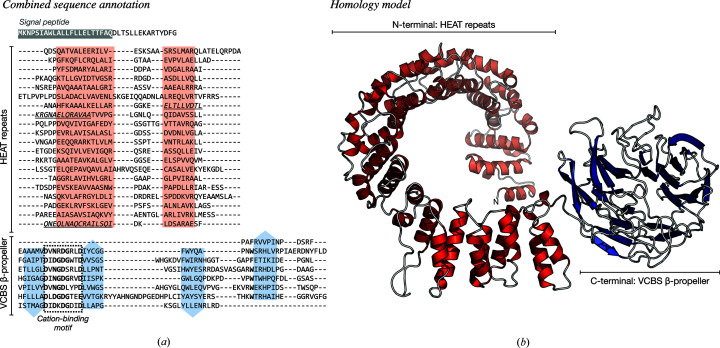
The domain organization and structural model of the hypothetical protein EHM23_20970. (*a*) In the sequence annotation, the N-terminal signal peptide detected by *SignalP* is colored gray and the alignments of repeats in the two domains are shown. Predicted α-helices are highlighted in red and β-strands in blue, and the repetitive, putative cation-binding motif in the β-propeller domain is highlighted by a dashed box. The region predicted to be a putative coiled coil by *PCOILS* is underlined. (*b*) A full-length homology model constructed with *MODELLER* is shown; secondary structure is colored as in (*a*).

To analyze the tandem repeats within the two domains of EHM23_20970 at the sequence level, we used *HHrepID* with default settings. We detected 20 short α-hairpins in the N-terminal region and seven four-stranded β-meanders in the C-terminal β-propeller region. These repeats are, however, quite degenerate and hard to find, especially in the propeller domain (Fig. 2 ▸ *a*), and therefore they had to be manually realigned using the structural model as a reference (Fig. 6 ▸ *a*). The obtained sequence alignments for the two regions highlight the presence of conserved sequence motifs (Fig. 1 ▸ *c*): while the HEAT repeats show a pattern of hydrophobic residues characteristic of amphiphilic α-helices, the β-propeller contains seven conserved D*x*DGDG*xx*D sequence motifs. A very similar aspartic acid-rich motif is characteristic of the VCBS superfamily of β-propeller-containing proteins, especially PVL lectins, where it is usually involved in binding cations; this motif is also found in α-integrin (Pereira & Lupas, 2021 ▸; Rigden & Galperin, 2004 ▸; Rigden *et al.*, 2011 ▸).

*PSI-BLAST* and *HHpred* searches for homologs of EHM23_20970 alerted us to the possible presence of putative coiled-coil regions, detected with *PCOILS*, and a signal peptide, detected with *SignalP* (Figs. 1 ▸ *a* and 1 ▸ *b*). While we could not detect coiled coils using the sensitive coiled-coil prediction method *DeepCoil* and manual inspection (Fig. 4 ▸), we detected a signal sequence using *Quick*2*D* (Fig. 5 ▸ *b*), *SignalP* and *TOPCONS*. Put together, our annotation suggests that the hypothetical protein EHM23_20970 is a secreted, two-domain protein, with N-terminal HEAT repeats and a C-terminal seven-bladed, PVL-like β-propeller with seven conserved cation-binding motifs. Given that HEAT repeats are usually involved in protein–protein interactions and PVL is a lectin (Yoshimura & Hirano, 2016 ▸; Cioci *et al.*, 2006 ▸), it is likely that EHM23_20970 is a secreted binder (perhaps a lectin) involved in a scaffolding role. However, it remains unclear whether it is a periplasmic protein or whether it is exported further across the outer membrane.

## 9. Summary   

The MPI Bioinformatics Toolkit provides easy and integrative access to a wide variety of bioinformatics tools and databases. It includes tools for the annotation of sequence features, the detection of remote homologs and the generation of homology models. Most tools within the Toolkit are interconnected, allowing the output of one to be forwarded as input to another. Starting from the amino-acid sequence of a hypothetical protein (EHM23_20970), the combination of these tools allowed us to predict that it contains two repetitive domains, which are likely to be involved in macromolecular binding, that it contains seven putative cation-binding sites and that it is likely to be transported across the inner membrane. Although no full-length homologs of known structure are presently available for this protein, we could build a preliminary three-dimensional model for it. This knowledge could now be used to design more streamlined experiments for its biochemical and biophysical characterization or to solve its structure using molecular replacement.

In addition to the tools described here, the Toolkit offers several other useful tools such as *CLANS* (Frickey & Lupas, 2004 ▸), which allows the generation of sequence-similarity networks (SSNs) for the visualization of relationships in large protein sequence sets (see Gabler *et al.*, 2020 ▸). Furthermore, we note that most of the analyses described in this article can also be performed using other web-based bioinformatics resources. For instance, the CBS (https://services.healthtech.dtu.dk/) and *PredictProtein* (Bernhofer *et al.*, 2021 ▸) servers are excellent resources for the prediction of sequence features in proteins, the NCBI *BLAST* (NCBI Resource Coordinators, 2018 ▸) and EBI *HMMER* (Potter *et al.*, 2018 ▸) servers for sequence-similarity searching, *EFI-EST* for the generation of SSNs (Zallot *et al.*, 2021 ▸) and the *SWISS-MODEL* server (Waterhouse *et al.*, 2018 ▸) for homology modeling. For *ab initio* structure prediction, we recommend the recently developed deep learning-based methods *AlphaFold* (Senior *et al.*, 2020 ▸; Jumper *et al.*, 2021 ▸; Tunyasuvunakool *et al.*, 2021 ▸; https://colab.research.google.com/github/deepmind/alphafold/blob/main/notebooks/AlphaFold.ipynb or https://github.com/sokrypton/ColabFold) and *RoseTTAFold* (Baek *et al.*, 2021 ▸; https://robetta.bakerlab.org), both of which promise to revolutionize the field of structural biology.

## References

[bb1] Almagro Armenteros, J. J., Tsirigos, K. D., Sønderby, C. K., Petersen, T. N., Winther, O., Brunak, S., von Heijne, G. & Nielsen, H. (2019). *Nat. Biotechnol.* **37**, 420–423. 10.1038/s41587-019-0036-z 30778233

[bb2] Altschul, S. F., Madden, T. L., Schäffer, A. A., Zhang, J., Zhang, Z., Miller, W. & Lipman, D. J. (1997). *Nucleic Acids Res.* **25**, 3389–3402. 10.1093/nar/25.17.3389 PMC146917 9254694

[bb3] Andrade, M. A., Perez-Iratxeta, C. & Ponting, C. P. (2001). *J. Struct. Biol.* **134**, 117–131. 10.1006/jsbi.2001.4392 11551174

[bb4] Andrade, M. A., Petosa, C., O’Donoghue, S. I., Müller, C. W. & Bork, P. (2001). *J. Mol. Biol.* **309**, 1–18. 10.1006/jmbi.2001.4624 11491282

[bb5] Baek, M., DiMaio, F., Anishchenko, I., Dauparas, J., Ovchinnikov, S., Lee, G. R., Wang, J., Cong, Q., Kinch, L. N., Schaeffer, R. D., Millán, C., Park, H., Adams, C., Glassman, C. R., DeGiovanni, A., Pereira, J. H., Rodrigues, A. V., van Dijk, A. A., Ebrecht, A. C., Opperman, D. J., Sagmeister, T., Buhlheller, C., Pavkov-Keller, T., Rathinaswamy, M. K., Dalwadi, U., Yip, C. K., Burke, J. E., Garcia, K. C., Grishin, N. V., Adams, P. D., Read, R. J. & Baker, D. (2021). *Science*, eabj8754.

[bb6] Bernhofer, M., Dallago, C., Karl, T., Satagopam, V., Heinzinger, M., Littmann, M., Olenyi, T., Qiu, J., Schütze, K., Yachdav, G., Ashkenazy, H., Ben-Tal, N., Bromberg, Y., Goldberg, T., Kajan, L., O’Donoghue, S., Sander, C., Schafferhans, A., Schlessinger, A., Vriend, G., Mirdita, M., Gawron, P., Gu, W., Jarosz, Y., Trefois, C., Steinegger, M., Schneider, R. & Rost, B. (2021). *Nucleic Acids Res.* **49**, W535–W540. 10.1093/nar/gkab354 PMC8265159 33999203

[bb7] Biegert, A. & Söding, J. (2008). *Bioinformatics*, **24**, 807–814. 10.1093/bioinformatics/btn039 18245125

[bb8] Chandonia, J.-M., Fox, N. K. & Brenner, S. E. (2019). *Nucleic Acids Res.* **47**, D475–D481. 10.1093/nar/gky1134 PMC6323910 30500919

[bb9] Cheng, H., Schaeffer, R. D., Liao, Y., Kinch, L. N., Pei, J., Shi, S., Kim, B.-H. & Grishin, N. V. (2014). *PLoS Comput. Biol.* **10**, e1003926. 10.1371/journal.pcbi.1003926 PMC4256011 25474468

[bb10] Cioci, G., Mitchell, E. P., Chazalet, V., Debray, H., Oscarson, S., Lahmann, M., Gautier, C., Breton, C., Perez, S. & Imberty, A. (2006). *J. Mol. Biol.* **357**, 1575–1591. 10.1016/j.jmb.2006.01.066 16497330

[bb11] Dalcin Martins, P., Danczak, R. E., Roux, S., Frank, J., Borton, M. A., Wolfe, R. A., Burris, M. N. & Wilkins, M. J. (2018). *Microbiome*, **6**, 138. 10.1186/s40168-018-0522-4 PMC6081815 30086797

[bb12] Delorenzi, M. & Speed, T. (2002). *Bioinformatics*, **18**, 617–625. 10.1093/bioinformatics/18.4.617 12016059

[bb13] Frickey, T. & Lupas, A. (2004). *Bioinformatics*, **20**, 3702–3704. 10.1093/bioinformatics/bth444 15284097

[bb14] Gabler, F., Nam, S.-Z., Till, S., Mirdita, M., Steinegger, M., Söding, J., Lupas, A. N. & Alva, V. (2020). *Curr. Protoc. Bioinform.* **72**, e108. 10.1002/cpbi.108 33315308

[bb15] Gruber, M., Söding, J. & Lupas, A. N. (2005). *Nucleic Acids Res.* **33**, W239–W243. 10.1093/nar/gki405 PMC1160166 15980460

[bb16] Gruber, M., Söding, J. & Lupas, A. N. (2006). *J. Struct. Biol.* **155**, 140–145. 10.1016/j.jsb.2006.03.009 16870472

[bb17] Haddad, Y., Adam, V. & Heger, Z. (2020). *PLoS Comput. Biol.* **16**, e1007449. 10.1371/journal.pcbi.1007449 PMC7117658 32240155

[bb18] Hayat, S., Peters, C., Shu, N., Tsirigos, K. D. & Elofsson, A. (2016). *Bioinformatics*, **32**, 1571–1573. 10.1093/bioinformatics/btw025 26794316

[bb19] Heffernan, R., Paliwal, K., Lyons, J., Singh, J., Yang, Y. & Zhou, Y. (2018). *J. Comput. Chem.* **39**, 2210–2216. 10.1002/jcc.25534 30368831

[bb20] Heinzinger, M., Elnaggar, A., Wang, Y., Dallago, C., Nechaev, D., Matthes, F. & Rost, B. (2019). *BMC Bioinformatics*, **20**, 723. 10.1186/s12859-019-3220-8 PMC6918593 31847804

[bb21] Hildebrand, A., Remmert, M., Biegert, A. & Söding, J. (2009). *Proteins*, **77**, 128–132. 10.1002/prot.22499 19626712

[bb22] Jamshidiha, M., Pérez-Dorado, I., Murray, J. W., Tate, E. W., Cota, E. & Read, R. J. (2019). *Acta Cryst.* D**75**, 342–353. 10.1107/S2059798318017825 PMC6450061 30950405

[bb23] Jernigan, K. K. & Bordenstein, S. R. (2015). *PeerJ*, **3**, e732. 10.7717/peerj.732 PMC4304861 25653910

[bb24] Johnson, L. S., Eddy, S. R. & Portugaly, E. (2010). *BMC Bioinformatics*, **11**, 431. 10.1186/1471-2105-11-431 PMC2931519 20718988

[bb25] Jones, D. T. (1999). *J. Mol. Biol.* **292**, 195–202. 10.1006/jmbi.1999.3091 10493868

[bb26] Jones, D. T. & Cozzetto, D. (2015). *Bioinformatics*, **31**, 857–863. 10.1093/bioinformatics/btu744 PMC4380029 25391399

[bb27] Jumper, J., Evans, R., Pritzel, A., Green, T., Figurnov, M., Ronneberger, O., Tunyasuvunakool, K., Bates, R., Žídek, A., Potapenko, A., Bridgland, A., Meyer, C., Kohl, S. A. A., Ballard, A. J., Cowie, A., Romera-Paredes, B., Nikolov, S., Jain, R., Adler, J., Back, T., Petersen, S., Reiman, D., Clancy, E., Zielinski, M., Steinegger, M., Pacholska, M., Berghammer, T., Bodenstein, S., Silver, D., Vinyals, O., Senior, A. W., Kavukcuoglu, K., Kohli, P. & Hassabis, D. (2021). *Nature*, https://doi.org/10.1038/s41586-021-03819-2.

[bb28] Kabsch, W. & Sander, C. (1983). *Biopolymers*, **22**, 2577–2637. 10.1002/bip.360221211 6667333

[bb29] Käll, L., Krogh, A. & Sonnhammer, E. L. L. (2004). *J. Mol. Biol.* **338**, 1027–1036. 10.1016/j.jmb.2004.03.016 15111065

[bb30] Karpenahalli, M. R., Lupas, A. N. & Söding, J. (2007). *BMC Bioinformatics*, **8**, 2. 10.1186/1471-2105-8-2 PMC1774580 17199898

[bb31] Klausen, M. S., Jespersen, M. C., Nielsen, H., Jensen, K. K., Jurtz, V. I., Sønderby, C. K., Sommer, M. O. A., Winther, O., Nielsen, M., Petersen, B. & Marcatili, P. (2019). *Proteins*, **87**, 520–527. 10.1002/prot.25674 30785653

[bb32] Kleywegt, G. J. (1996). *Acta Cryst.* D**52**, 842–857. 10.1107/S0907444995016477 15299650

[bb33] Krogh, A., Larsson, B., von Heijne, G. & Sonnhammer, E. L. (2001). *J. Mol. Biol.* **305**, 567–580. 10.1006/jmbi.2000.4315 11152613

[bb34] Ladunga, I. (2017). *Curr. Protoc. Bioinform.* **59**, 3. 10.1002/cpbi.34 28902395

[bb35] Li, C., Ching Han Chang, C., Nagel, J., Porebski, B. T., Hayashida, M., Akutsu, T., Song, J. & Buckle, A. M. (2016). *Brief. Bioinform.* **17**, 270–282. 10.1093/bib/bbv047 PMC6078162 26177815

[bb36] Liu, Y., Schmidt, B. & Maskell, D. L. (2010). *Bioinformatics*, **26**, 1958–1964. 10.1093/bioinformatics/btq338 20576627

[bb37] Ludwiczak, J., Winski, A., Szczepaniak, K., Alva, V. & Dunin-Horkawicz, S. (2019). *Bioinformatics*, **35**, 2790–2795. 10.1093/bioinformatics/bty1062 30601942

[bb38] Lupas, A., Van Dyke, M. & Stock, J. (1991). *Science*, **252**, 1162–1164. 10.1126/science.252.5009.1162 2031185

[bb39] Lupas, A. N. & Bassler, J. (2017). *Trends Biochem. Sci.* **42**, 130–140. 10.1016/j.tibs.2016.10.007 27884598

[bb40] Lupas, A. N., Bassler, J. & Dunin-Horkawicz, S. (2017). *Subcell. Biochem.* **82**, 95–129. 10.1007/978-3-319-49674-0_4 PMC7122542 28101860

[bb41] Madeira, F., Park, Y. M., Lee, J., Buso, N., Gur, T., Madhusoodanan, N., Basutkar, P., Tivey, A. R. N., Potter, S. C., Finn, R. D. & Lopez, R. (2019). *Nucleic Acids Res.* **47**, W636–W641. 10.1093/nar/gkz268 PMC6602479 30976793

[bb42] Madeo, G., Savojardo, C., Martelli, P. L. & Casadio, R. (2020). *J. Mol. Biol.* **433**, 166729. 10.1016/j.jmb.2020.166729 33972021

[bb43] Marcotte, E. M., Pellegrini, M., Yeates, T. O. & Eisenberg, D. (1999). *J. Mol. Biol.* **293**, 151–160. 10.1006/jmbi.1999.3136 10512723

[bb44] Mirdita, M., von den Driesch, L., Galiez, C., Martin, M. J., Söding, J. & Steinegger, M. (2017). *Nucleic Acids Res.* **45**, D170–D176. 10.1093/nar/gkw1081 PMC5614098 27899574

[bb45] Mistry, J., Chuguransky, S., Williams, L., Qureshi, M., Salazar, G. A., Sonnhammer, E. L. L., Tosatto, S. C. E., Paladin, L., Raj, S., Richardson, L. J., Finn, R. D. & Bateman, A. (2020). *Nucleic Acids Res.* **49**, D412–D419. 10.1093/nar/gkaa913 PMC7779014 33125078

[bb46] NCBI Resource Coordinators (2018). *Nucleic Acids Res.* **46**, D8–D13. 10.1093/nar/gkx1095 PMC5753372 29140470

[bb47] Pereira, J. & Lupas, A. N. (2021). *Bioinformatics*, **36**, 5618–5622. 10.1093/bioinformatics/btaa1085 PMC8023676 33416871

[bb48] Peters, M., Neumann, M., Iyyer, M., Gardner, M., Clark, C., Lee, K. & Zettlemoyer, L. (2018). *Proceedings of the 2018 Conference of the North American Chapter of the Association for Computational Linguistics: Human Language Technologies, Volume 1 (Long Papers)*, edited by M. Walker, H. Ji & A. Stent, pp. 2227–2237. Stroudsburg: Association for Computational Linguistics.

[bb49] Potter, S. C., Luciani, A., Eddy, S. R., Park, Y., Lopez, R. & Finn, R. D. (2018). *Nucleic Acids Res.* **46**, W200–W204. 10.1093/nar/gky448 PMC6030962 29905871

[bb50] Prakash, A., Jeffryes, M., Bateman, A. & Finn, R. D. (2017). *Curr. Protoc. Bioinform.* **60**, 3. 10.1002/cpbi.40 29220076

[bb51] Remmert, M., Biegert, A., Hauser, A. & Söding, J. (2012). *Nat. Methods*, **9**, 173–175. 10.1038/nmeth.1818 22198341

[bb52] Remmert, M., Biegert, A., Linke, D., Lupas, A. N. & Söding, J. (2010). *Mol. Biol. Evol.* **27**, 1348–1358. 10.1093/molbev/msq017 20106904

[bb53] Remmert, M., Linke, D., Lupas, A. N. & Söding, J. (2009). *Nucleic Acids Res.* **37**, W446–W451. 10.1093/nar/gkp325 PMC2703889 19429691

[bb54] Rigden, D. J. & Galperin, M. Y. (2004). *J. Mol. Biol.* **343**, 971–984. 10.1016/j.jmb.2004.08.077 15476814

[bb55] Rigden, D. J., Woodhead, D. D., Wong, P. W. H. & Galperin, M. Y. (2011). *PLoS One*, **6**, e21507. 10.1371/journal.pone.0021507 PMC3123361 21720552

[bb56] Ruf, A., Tetaz, T., Schott, B., Joseph, C. & Rudolph, M. G. (2016). *Acta Cryst.* D**72**, 1212–1224. 10.1107/S2059798316016715 PMC5108348 27841754

[bb57] Senior, A. W., Evans, R., Jumper, J., Kirkpatrick, J., Sifre, L., Green, T., Qin, C., Žídek, A., Nelson, A. W. R., Bridgland, A., Penedones, H., Petersen, S., Simonyan, K., Crossan, S., Kohli, P., Jones, D. T., Silver, D., Kavukcuoglu, K. & Hassabis, D. (2020). *Nature*, **577**, 706–710. 10.1038/s41586-019-1923-7 31942072

[bb58] SIB Swiss Institute of Bioinformatics Members (2016). *Nucleic Acids Res.* **44**, D27–D37. 10.1093/nar/gkv1310 PMC4702916 26615188

[bb59] Söding, J. (2005). *Bioinformatics*, **21**, 951–960. 10.1093/bioinformatics/bti125 15531603

[bb60] Söding, J. & Lupas, A. N. (2003). *Bioessays*, **25**, 837–846. 10.1002/bies.10321 12938173

[bb61] Steinegger, M., Meier, M., Mirdita, M., Vöhringer, H., Haunsberger, S. J. & Söding, J. (2019). *BMC Bioinformatics*, **20**, 473. 10.1186/s12859-019-3019-7 PMC6744700 31521110

[bb62] Terwilliger, T. C. (2002). *Acta Cryst.* D**58**, 2082–2086. 10.1107/S0907444902016360 PMC2745884 12454468

[bb63] Touw, W. G., Baakman, C., Black, J., te Beek, T. A. H., Krieger, E., Joosten, R. P. & Vriend, G. (2015). *Nucleic Acids Res.* **43**, D364–D368. 10.1093/nar/gku1028 PMC4383885 25352545

[bb64] Tsirigos, K. D., Peters, C., Shu, N., Käll, L. & Elofsson, A. (2015). *Nucleic Acids Res.* **43**, W401–W407. 10.1093/nar/gkv485 PMC4489233 25969446

[bb65] Tunyasuvunakool, K., Adler, J., Wu, Z., Green, T., Zielinski, M., Žídek, A., Bridgland, A., Cowie, A., Meyer, C., Laydon, A., Velankar, S., Kleywegt, G. J., Bateman, A., Evans, R., Pritzel, A., Figurnov, M., Ronneberger, O., Bates, R., Kohl, S. A. A., Potapenko, A., Ballard, A. J., Romera-Paredes, B., Nikolov, S., Jain, R., Clancy, E., Reiman, D., Petersen, S., Senior, A. W., Kavukcuoglu, K., Birney, E., Kohli, P., Jumper, J. & Hassabis, D. (2021). *Nature*, https://doi.org/10.1038/s41586-021-03828-1.

[bb66] Waterhouse, A., Bertoni, M., Bienert, S., Studer, G., Tauriello, G., Gumienny, R., Heer, F. T., de Beer, T. A. P., Rempfer, C., Bordoli, L., Lepore, R. & Schwede, T. (2018). *Nucleic Acids Res.* **46**, W296–W303. 10.1093/nar/gky427 PMC6030848 29788355

[bb67] Webb, B. & Sali, A. (2021). *Methods Mol. Biol.* **2199**, 239–255. 10.1007/978-1-0716-0892-0_14 33125654

[bb68] Yang, J., Anishchenko, I., Park, H., Peng, Z., Ovchinnikov, S. & Baker, D. (2020). *Proc. Natl Acad. Sci. USA*, **117**, 1496–1503. 10.1073/pnas.1914677117 PMC6983395 31896580

[bb69] Yang, M., Derbyshire, M. K., Yamashita, R. A. & Marchler-Bauer, A. (2020). *Curr. Protoc. Bioinform.* **69**, e90. 10.1002/cpbi.90 PMC7378889 31851420

[bb70] Yoshimura, S. H. & Hirano, T. (2016). *J. Cell Sci.* **129**, 3963–3970. 10.1242/jcs.185710 27802131

[bb71] Zallot, R., Oberg, N. & Gerlt, J. A. (2021). *Curr. Opin. Biotechnol.* **69**, 77–90. 10.1016/j.copbio.2020.12.004 PMC8238782 33418450

[bb72] Zimmermann, L., Stephens, A., Nam, S.-Z., Rau, D., Kübler, J., Lozajic, M., Gabler, F., Söding, J., Lupas, A. N. & Alva, V. (2018). *J. Mol. Biol.* **430**, 2237–2243. 10.1016/j.jmb.2017.12.007 29258817

